# Assessment of bidirectional relationships between brain imaging-derived phenotypes and stroke: a Mendelian randomization study

**DOI:** 10.1186/s12916-023-02982-9

**Published:** 2023-07-25

**Authors:** Ke Yu, Xiao-Feng Chen, Jing Guo, Sen Wang, Xiao-Ting Huang, Yan Guo, Shan-Shan Dong, Tie-Lin Yang

**Affiliations:** Key Laboratory of Biomedical Information Engineering of Ministry of Education, Biomedical Informatics & Genomics Center, School of Life Science and Technology, Xi’an Jiaotong University, Xi’an, 710049 People’s Republic of China

**Keywords:** Stroke, IDPs, Causal association, Mendelian randomization

## Abstract

**Background:**

Stroke is a major cause of mortality and long-term disability worldwide. Whether the associations between brain imaging-derived phenotypes (IDPs) and stroke are causal is uncertain.

**Methods:**

We performed two-sample bidirectional Mendelian randomization (MR) analyses to explore the causal associations between IDPs and stroke. Summary data of 587 brain IDPs (up to 33,224 individuals) from the UK Biobank and five stroke types (sample size range from 301,663 to 446,696, case number range from 5,386 to 40,585) from the MEGASTROKE consortium were used.

**Results:**

Forward MR indicated 14 IDPs belong to projection fibers or association fibers were associated with stroke. For example, higher genetically determined mean diffusivity (MD) in the right external capsule was causally associated with an increased risk of small vessel stroke (IVW OR = 2.76, 95% CI 2.07 to 3.68, *P* = 5.87 × 10^−12^). Reverse MR indicated that genetically determined higher risk of any ischemic stroke was associated with increased isotropic or free water volume fraction (ISOVF) in body of corpus callosum (IVW *β* = 0.23, 95% CI 0.14 to 0.33, *P* = 3.22 × 10^−7^). This IDP is a commissural fiber and it is not included in the IDPs identified by forward MR.

**Conclusions:**

We identified 14 IDPs with statistically significant evidence of causal effects on stroke or stroke subtypes. We also identified potential causal effects of stroke on one IDP of commissural fiber. These findings might guide further work toward identifying preventative strategies at the brain imaging levels.

**Supplementary Information:**

The online version contains supplementary material available at 10.1186/s12916-023-02982-9.

## Background

Stroke is defined as an abrupt neurological outburst caused by impaired perfusion through the blood vessels to the brain. It is a major cause of mortality and long-term disability worldwide. Globally, more than 100 million people have been reported to suffer from stroke [1].

Stroke can be characterized by physiological brain changes which can be measured noninvasively using magnetic resonance imaging (MRI) [2]. Indeed, many observational studies have been performed to explore the relationships between brain imaging-derived phenotypes (IDPs) and stroke. Researchers have tried to investigate whether IDPs could potentially affect the risk of stroke. For example, fractional anisotropy (FA) is a commonly used MRI measure of the anisotropic diffusion degree with values between one (intact white matter) and zero (disrupted white matter). A follow-up study for 4259 subjects found that lower FA increased the risk of stroke, independent of other risk factors [3]. Several prospective studies [4–6] conducted on a range of different populations found that white matter lesions are correlated with increased risk of stroke. In addition to studies in pre-stroke subjects, many studies have also used MRI to assess brain changes in patients after stroke. For example, in post-stroke patients, reduced FA within the ischemic lesion or in the corticospinal tract remote from the lesion was observed [7–9]. Moreover, lower FA values were associated with greater motor deficit and worse motor recovery [10, 11]. However, conventional observational studies are more likely influenced by residual confounding. Therefore, whether the associations between IDPs and stroke are causal is uncertain.

Mendelian randomization (MR) is a useful genetic epidemiology study design using genetic variants as instrumental variables (IVs) to investigate whether the exposure is causally related to a medically relevant disease risk [12]. Since inherent genetic variants are not susceptible to environmental variables, the MR design can avoid the potential confounding factors that are common in conventional observational studies [13]. In this study, we used two-sample MR to systematically investigate the bidirectional causal associations between 587 IDPs and stroke. Analyses were performed for 5 stroke types, including any stroke (AS), which comprises ischemic stroke, intracerebral hemorrhage, and stroke of undetermined type; any ischemic stroke (AIS) regardless of subtype; and three ischemic stroke subtypes: large-artery atherosclerotic stroke (LAS), cardioembolic stroke (CES), small-vessel stroke (SVS). We chose these 5 types according to the common etiological subtypes of stroke [14]. Our results might offer new insights into the prevention, diagnosis and treatment for stroke.

## Methods

### Overview of the study

**Fig. 1:**
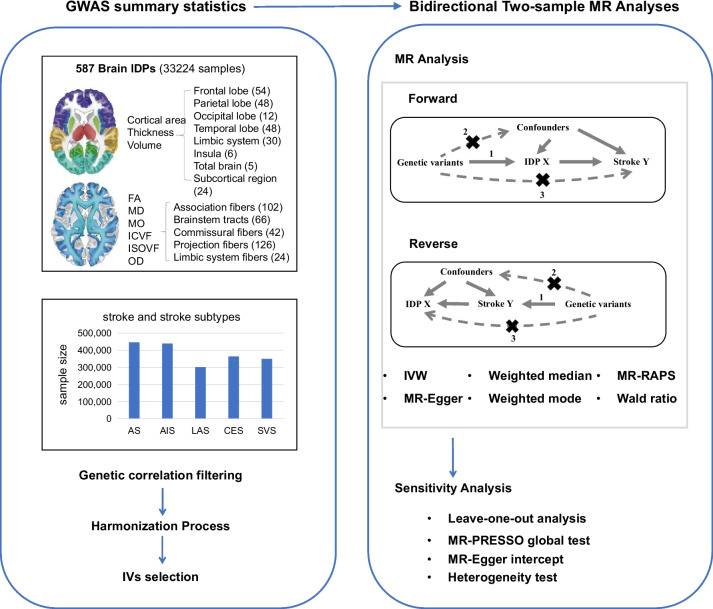
Workflow of the causal inference between IDPs and stroke. AS, any stroke; AIS, any ischemic stroke; CES, cardioembolic stroke; LAS, large artery stroke; MAF, minor allele frequency; LD, linkage disequilibrium; SVS, small vessel stroke

The study design is shown in Fig. 1. We obtained the GWAS summary data of 587 IDPs derived from the UK Biobank. Summary data of five stroke traits were also collected (Additional file 1: Table S1). Prior to MR analyses, genetic correlation analyses were performed. Then, we conducted bidirectional two-sample MR analysis between the exposure-outcome pairs with genetic correlation.

### Data sets of brain IDPs

GWAS summary statistics of IDPs processed with a sample size of 33,224 individuals of European ancestry from the UK Biobank release 2020 [15]. We focused on the causal associations between brain structural IDPs and stroke. There are 2146 brain structural IDPs from the original UKB release. To make sure our results are reliable and reproducible, we filtered the IDPs using the following process: First, some regions might be measured more than one time with different tools, resulting in redundant IDPs. We only kept the IDPs measured by the most commonly used tools (FreeSurfer [16], FIRST [17], tract-based spatial statistics [18] and probabilistic tractography [19]) and removed 1201 redundant IDPs. Second, we removed 358 IDPs derived from small brain areas which do not have strong contrast on MR images (can’t be reliably segmented and measured). Finally, 587 IDPs were selected [20].

These IDPs were derived from two measurements: structural MRI (sMRI) and diffusion MRI (dMRI). The sMRI can capture the variations of brain anatomy while dMRI measures structural connectivity, which describes neural fiber physical connections between brain regions. We further classified the IDPs into 13 regional categories and 9 measures. As shown in Fig. 1, these regions included 8 regions for brain anatomy (such as frontal lobe and parietal lobe) and 5 regions for structural connectivity (such as association fibers and brainstem tracts). For brain anatomy, 3 measures including area, thickness, and volume were obtained. For structural connectivity, 6 measures including FA, mean diffusivity (MD), diffusion tensor mode (MO), intracellular volume fraction (ICVF), isotropic or free water volume fraction (ISOVF), and orientation dispersion index (OD) were obtained. Details of these IDPs is shown in the website: http://www.bigc.online/strokeMR/IDP.html.

### GWAS datasets for stroke

We used the GWAS summary statistics of five stroke types (including AS, AIS, SVS, CES, and LAS) with a sample size of ranged from 301,663 to 446,696 individuals (case number range from 5386 to 40,585) of European ancestry from the MEGASTROKE consortium (https://www.megastroke.org/) release 2018 [14]. Detail information of these datasets is shown in Additional file 1: Table S1.

### Harmonized process of datasets

We removed SNPs with minor allele frequencies (MAF) < 0.01 in the GWAS datasets. For all stroke GWAS summary statistics, the OR value was converted to log odds. We removed SNPs in regions of long-range, high linkage disequilibrium in the human genome, such as the human major histocompatibility complex (MHC) region. The region list was obtained from the web site: https://genome.sph.umich.edu/wiki/Regions_of_high_linkage_disequilibrium_(LD)#cite_note-3 [21].

### Genetic correlation analysis

We performed a genetic correlation analysis prior to MR analyses to strengthen the understanding of the relationships between complex traits [22]. We used linkage disequilibrium score regression (LDSC) to screen the evidence of genetic correlation between IDPs and stroke for subsequent MR analysis [23]. We used *P* < 0.05 as the threshold to preserve all IDP-stroke pairs with suggestive evidence, that is, only the IDP-stroke pairs with nominal statistical significance in LDSC were used in subsequent bi-directional MR analyses. We chose this analysis strategy in refer to previous studies [20, 24].

### MR analysis

#### IV selection

We used the same criteria for the bidirectional Two-Sample MR analysis when we select the IVs. Firstly, we used the clumping algorithm in PLINK (https://www.cog-genomics.org/plink/1.9/) [25] to select independent SNPs for each exposure (*r*^2^ threshold = 0.001, window size = 1 Mb and *P* < 5 × 10^−8^). The 1000G European data (phase 3) were used as the reference for LD estimation [26]. Secondly, if one selected SNP in the first step was not present in outcome data, we used its proxy SNP with *r*^2^ > 0.8 instead. The proxy SNP must also be associated with exposure (*P* < 5 × 10^−8^). If more than one proxy SNPs were available, we chose the one with the maximum *r*^2^ and minimum *P* value associated with the exposure.

#### IV quality control

For the IVs, three key assumptions must hold: (1) the selected IVs must be associated with the exposure (relevance assumption); (2) the selected IVs are not associated with potential confounders (independence assumption), and (3) the IVs affect the outcome only through their effect on the exposure (exclusion restriction assumption). We used RadialMR package [27] to remove pleiotropic SNPs. We also looked up each IV and its proxies in the PhenoScanner GWAS database (http://phenoscanner.medschl.cam.ac.uk) to assess any associations with potential confounders (smoking, drinking, blood pressure, socioeconomic status and education) [28]. The proxy selection was performed directly by the PhenoScanner database. The LD threshold was set as *r*^2^ > 0.8, and the genome version we used was GRCh37. SNPs associated with potential confounders were removed. The remaining SNPs were used to perform MR analysis. We used MR Steiger filtering to check whether the MR analysis estimates assessed the true causal direction [29].

### Bidirectional two-sample MR analyses

We used two-sample MR analyses to explore the causal relationships between brain IDPs and stroke. We selected IVW regression with multiplicative random effects model as the primary method for causal inference [30], that is, the evidence of statistically significant causal effect was based on the IVW *P*-value. To complement and enhance the reliability of the results, we conducted four other MR methods. MR-Egger method estimates the causal effect through the slope coefficient of the Egger regression, which provides a more robust estimate even if none of the IVs are invalid [31]. Weighted median method can even protect against up to 50% of invalid IVs [32]. Weighted mode method provides consistent estimates when the relaxed IV assumption has less bias and a lower type-I error rate [33]. MR-RAPS accounts for systematic and idiosyncratic pleiotropy and can provide a robust inference for MR analysis with many weak instruments [34]. Wald ratio for MR analysis was used when only 1 genetic instrument was available [35]. All these methods were implemented in the TwoSampleMR R package [36]. We extended the above MR analysis to a bidirectional causal inference between IDPs and stroke. Forward MR analyses were carried out with brain IDPs as exposures and stroke as outcomes. Conversely, reverse MR analyses were carried out with stroke as exposures and brain IDPs as outcomes.

### Sensitivity analysis

We conducted Bonferroni correction across all MR tests. For the significant MR results after Bonferroni correction (*P* < 5.91 × 10^−5^ (0.05/423/2), 423 denotes the number of IDP-stroke pairs, 2 denotes forward and reverse MR tests), we further performed sensitivity analysis. First, we performed leave-one-out analysis to check whether the causal association was obviously driven by a single SNP (*P* value < 0.05 was regarded as an outlier). Second, we conducted MR-PRESSO to detect the presence of horizontal pleiotropy (*P* value < 0.05) [37]. Third, we executed MR-Egger regression to examine the potential bias of directional pleiotropy [31]. The intercept in the Egger regression indicates the mean pleiotropic effect of all genetic variants, which is interpreted as evidence of directional pleiotropy when the value differs from zero (*P* value < 0.05). Cochran’s *Q* and Rucker’s *Q*′ statistics were also calculated to check for the presence of heterogeneity for IVW and MR-Egger method [27], respectively.

### Data availability

GWAS statistics of brain IDPs were collected from BIG40 web browser (https://open.win.ox.ac.uk/ukbiobank/big40). GWAS statistics of stroke were obtained from MEGASTROKE consortium (https://www.megastroke.org/).

### Code availability

All software packages we used in the study are publicly available, and the download links are included in the “Methods” section. We have put our code on the website: http://www.bigc.online/strokeMR/code.html.

## Results

### Genetic correlation

Before MR analysis, we implemented the LDSC analysis to examine genetic correlation between 587 IDPs and stroke. Genetic correlations on 423 IDP-stroke pairs were detected (*P* value < 0.05, Additional file 1: Table S2). Therefore, the significant threshold for our bidirectional MR analyses after multiple testing correction using the Bonferroni method was set as *P* < 5.91 × 10^−5^ (0.05/423/2).

### IV selection

After linkage disequilibrium (LD) pruning, we used 1000 Genomes as reference to select conditionally independent single-nucleotide polymorphisms (SNPs) with plink. Outlier IVs (Additional file 1: Tables S3 and S4) detected by the RadialMR method were removed from MR analysis. IVs associated with potential confounders were also removed (Additional file 1: Tables S5 and S6).

For the 423 IDP-stroke pairs with genetic correlation, we performed MR analysis only for the pairs whose exposure has at least one qualified IV. After removing the pairs with no qualified IV, there were 406 and 301 pairs for forward and reverse MR analysis, respectively. As shown in Table S7 and S8, the *F*-statistic values were all ≥ 30, suggesting the potential for weak instrumental bias to be low. In forward MR analysis, the variance explained by the selected variants for IDPs ranged from 0.10 to 7.03%. In reverse MR, the variance explained by the selected variants for strokes ranged from 0.12 to 2.08%. We used MR Steiger filtering to test whether the MR estimates assessed the true causal direction. As shown in Additional file 1: Tables S7 and S8, the IVs did explain more variance for the exposure than for the outcome.

The statistical power based on the sample size for the MR test showed that we had 80% power at a significance level of 0.05 to detect a minimum odds ratio (OR) of > 1.07 or < 0.94 in forward MR analyses and a minimum *β* of > 0.11 or < − 0.11 in reverse MR analyses.

### Forward MR: the putative causal effects of IDP on stroke

**Fig. 2:**
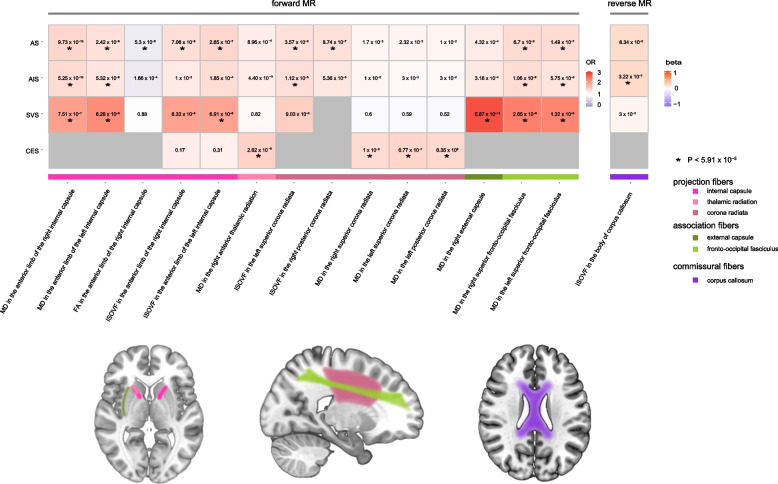
Causal associations between IDPs and stroke in the forward MR and reverse MR. The heatmap plot shows the causal associations between IDPs with at least one significant MR signals in forward and reverse MR and stroke. The pattern diagram in the bottom shows the brain anatomical region of corresponding IDPs. AS, any stroke; AIS, any ischemic stroke; CES, cardioembolic stroke; FA, fractional anisotropy; ISOVF, isotropic volume fraction; MD, mean diffusivity; SVS, small vessel stroke

**Fig. 3:**
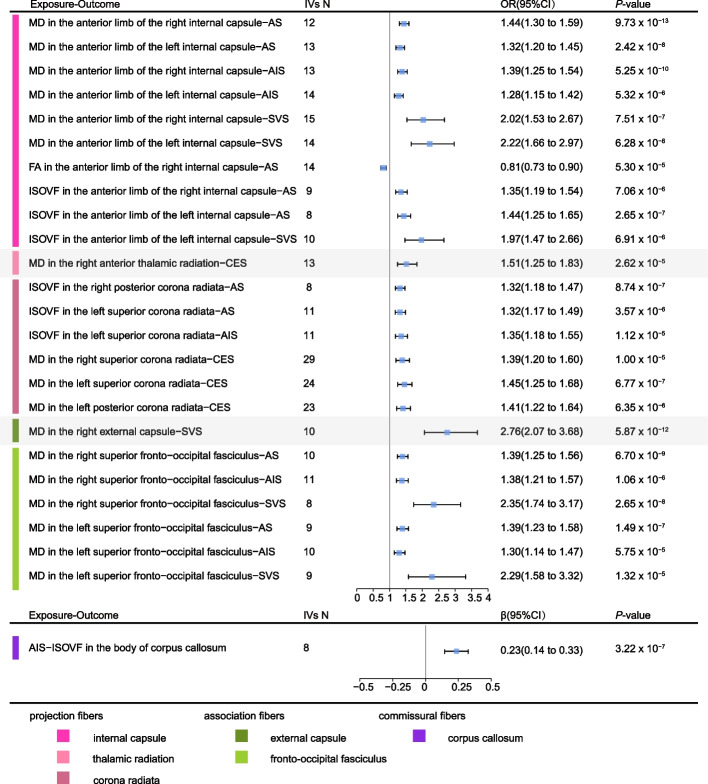
Causal associations in the forward and reverse MR using the MR-IVW method. The forest plot shows the significant causal associations with *P* value < 5.91 × 10^−5^ and the estimated OR (or beta) with 95% confidence intervals (CI). AS, any stroke; AIS, any ischemic stroke; CES, cardioembolic stroke; FA, fractional anisotropy; ISOVF, isotropic volume fraction; MD, mean diffusivity; SVS, small vessel stroke

As shown in Additional file 1: Table S9, there were 24 statistically significant causal relationships between 14 IDPs and stroke (Figs. 2 and 3). These IDPs are derived from projection fibers and association fibers. The scatter plots are shown in Additional file 2: Figure S1.

#### The putative causal effects of projection fibers on stroke

As shown in Figs. 2 and 3, and Additional file 1: Table S9, we observed that 11 IDPs which are projection fibers were causally associated with stroke. The projection fibers consist of efferent and afferent fibers uniting the cortex with the lower parts of the brain and with the spinal cord.

The anterior limb of internal capsule is a white matter structure which carries brainstem fibers and thalamic from prefrontal cortical regions. In this region, we find 10 statistically significant causal relationships. For example, one SD (3.76 × 10^−5^ AU) increase in the MD of the right anterior limb of internal capsule was associated with 44% higher odds of AS (OR = 1.44, 95% CI 1.30 to 1.59, *P* = 9.73 × 10^−13^), 39% higher odds of AIS (OR = 1.39, 95% CI 1.25 to 1.54, *P* = 5.25 × 10^−10^), and 102% higher odds of SVS (OR = 2.02, 95% CI 1.53 to 2.67, *P* = 7.51 × 10^−7^). Similar results were observed in the MD of the left anterior limb of internal capsule. MD is an indicator of the average mobility of water molecules. Consistently, we observed negative association between FA of the right anterior limb of internal capsule and the risk of AS (IVW OR = 0.81, 95% CI 0.73 to 0.90, *P* = 5.30 × 10^−5^). FA is a measurement of the degree of anisotropic diffusion. Generally, in damaged structurally organized tissue (e.g., white matter tracts), increased MD and decreased FA will be observed due to loss of the directionality of diffusion. In addition to MD and FA, we also observed positive associations between ISOVF of the anterior limb of internal capsule and the risk of AS or SVS (Figs. 2 and 3). ISOVF is an indicator of volume fraction of extracellular isotropic free water.

The anterior thalamic radiations connect the anterior and midline nuclear groups of the thalamus with the frontal lobe through the anterior thalamic peduncle and the anterior limb of the internal capsule. In this region, one SD (4.21 × 10^−5^ AU) increase in the MD of the tract right anterior thalamic radiation was associated with 51% upper odds of CES (OR = 1.51, 95% CI 1.25 to 1.83, *P* = 2.62 × 10^−5^).

The corona radiata is a key white matter structure that consists of afferent and efferent fibers that connect the cerebral cortex and the brain stem. In this region, we find 6 statistically significant causal relationships. One SD (0.02 AU) increase in the ISOVF of the left superior corona radiata was positively associated with 32% higher odds of AS (OR = 1.32, 95% CI 1.17 to 1.49, *P* = 3.57 × 10^−6^) and 35% higher odds of AIS (OR = 1.35, 95% CI 1.18 to 1.55, *P* = 1.12 × 10^−5^). Similarly, one SD (0.02 AU) increase in ISOVF of the right posterior corona radiata was associated with 32% higher odds of AS (OR = 1.32, 95% CI 1.18 to 1.47, *P* = 8.74 × 10^−7^). Specifically, we observed that MD of the left posterior corona radiata (OR = 1.41, 95% CI 1.22 to 1.64, *P* = 6.35 × 10^−6^), right superior corona radiata (OR = 1.39, 95% CI 1.2 to 1.6, *P* = 1.00 × 10^−5^), and left superior corona radiata (OR = 1.45, 95% CI 1.25 to 1.68, *P* = 6.77 × 10^−7^) were all positively associated with the risk of CES.

#### The putative causal effects of association fibers on stroke

As shown in Figs. 2 and 3, and Additional file 1: Table S9, we observed that 3 IDPs which are association fibers were causally associated with stroke. Association fibers are the fibers connecting different cortical areas of the same side to one another.

The external capsule is a series of white matter tracts in the brain situated between the putamen and claustrum. It is composed of claustrocortical fibers dorsally and the combined mass of the uncinate fasciculus and inferior frontal occipital fasciculus ventrally. In this region, one SD (3.74 × 10^−5^ AU) increase in the MD of the right external capsule was associated with 176% upper odds of SVS risk (IVW OR = 2.76, 95% CI 2.07 to 3.68, *P* = 5.87 × 10^−12^).

The superior fronto-occipital fasciculus connecting the frontal, occipital, and parietal lobe is an association fiber tract 2–3 mm in diameter. In this region, one SD (7.43 × 10^−5^ AU) increase in the MD of the right superior fronto-occipital fasciculus was associated with 39% higher odds of AS risk (IVW OR = 1.39, 95% CI 1.25 to 1.56, *P* = 6.70 × 10^−9^), 38% higher odds of AIS risk (IVW OR = 1.38, 95% CI 1.21 to 1.57, *P* = 1.06 × 10^−6^), and 135% upper odds of SVS risk (IVW OR = 2.35, 95% CI 1.74 to 3.17, *P* = 2.65 × 10^−8^). Similar results were observed in the MD of the left superior fronto-occipital fasciculus (Figs. 2 and 3).

### Reverse MR: the putative causal effects of stroke on IDPs

As shown in Figs. 2 and 3, and Additional file 1: Table S10, we did not detect significant causal effects of the risk of stroke on IDPs detected in the forward MR analyses. However, we found evidence that genetically determined higher risk of AIS was associated with increased ISOVF in body of corpus callosum (*β* = 0.22, 95% CI 0.14 to 0.33, *P* = 3.22 × 10^−7^, Figs. 2 and 3), which is a commissural fiber. The scatter plot is shown in Additional file 2: Figure S2. Commissural fibers connect an area in one hemisphere with an area in the opposite hemisphere and the corpus callosum is the largest commissural fiber. It allows us to perceive depth and enables the two sides of our brain to communicate.

### Sensitivity analyses

We performed sensitivity analyses to verify the results obtained with bidirectional MR. First, leave-one-out analyses showed that no single SNP drove the causal estimates (Additional file 2: Figure S3 and S4). Second, MR-Egger intercepts of all associations were close to zero, suggesting that no significant pleiotropy was detected (Additional file 1: Table S11-12). Additionally, Cochran’s *Q* and Rucker’s *Q*′ statistics were calculated to check for the presence of heterogeneity for IVW and MR-Egger method, respectively. As shown in Additional file 1: Table S11-12, no significant evidence of heterogeneity was detected.

## Discussion

Observational studies have reported that IDPs are associated with stroke; however, whether the relationships are causal is uncertain. In the present study, we performed bidirectional two-sample MR analyses to systematically investigate the causal associations between 587 IDPs and stroke. We identified 14 IDPs with statistically significant evidence of potential causal effects on stroke. We also identified potential causal effects of stroke on one IDP of commissural fiber.

In the forward MR analysis, we observed that 14 IDPs were causally associated with stroke. These IDPs were derived from projection or association fibers. Of note, these IDPs included 9 MD values and 1 FA value in different regions. As we mentioned in the results section, both measures are generally used to track structural integrity. Increased MD and decreased FA will be observed in damaged structurally organized tissue (e.g., white matter tracts). The MR results showed that higher genetically determined MD (or lower genetically determined FA) was causally associated with increased risk of AS, suggesting that disrupted integrity of the projection or association fibers is a potential risk factor for stroke. Changes in white matter microstructural integrity have been shown to precede irreversible white matter lesions [38], which has been widely reported as an important risk factor for stroke [4–6]. A recent study reported that white matter lesions showed significantly lower blood flow, blood volume, and capillary metabolic rate of oxygen [39]. This might increase the risk of thrombus and further lead to ischemic stroke which typically begins with an acute phase in which ischemia results from a thrombus that lodges in a cerebral blood vessel. Further studies are needed to clarify the underlying physiological mechanism. Consistent with our results, a population-based study found that both lower FA and higher MD increased risk of stroke, independent of other risk factors [3]. Another study carried out in a longitudinal cohort of 800 community-dwelling adults found that lower FA was associated with higher risk of stroke [40]. Taking white matter lesions into consideration could improve the performance of stroke risk prediction models [41].

The forward MR results showed that some IDPs were only associated with specific stroke subtypes. For instance, MD in the right external capsule was only positively associated with SVS. Consistent with our results, a previous study [42] has observed that compared with healthy controls, MD value in external capsule of cerebral autosomal dominant arteriopathy with subcortical infarcts and leukoencephalopathy (CADASIL, a common heritable cause of SVS) patients was significantly increased. White matter hyperintensities in this region or external capsule lesion in CADASIL patients were also reported in other studies [43, 44]. However, reports about the association between IDPs derived from external capsule and other stroke types are still limited. These results indicated that IDPs in specific regions might have different effects on different stroke subtypes.

In reverse MR analysis, we observed genetically determined higher risk of AIS was associated with increased ISOVF in body of corpus callosum. Corpus callosum is the largest commissural structure consisting of white matter tracts that connect the cerebral hemispheres according to an anterior–posterior topographical organization [45]. Higher ISOVF indicated increased extracellular water volume, which is expected in neuroinflammatory states [46]. Therefore, this reverse MR result suggested that higher risk of ischemic stroke might lead to the sequential injury in corpus callosum. In ischemic stroke, the ischemic area turns into a necrotic core. Degeneration of the distal axons may occur both near and far from the ischemic bed [47]. Consistent with our findings, a previous study [48] has observed damage of fiber tracts with their accompanying myelin sheaths in the nonischemic corpus callosum in the middle cerebral artery occlusion rat model. Degeneration of corpus callosum has also been observed in post-stroke patients in population-based studies [49, 50].

The significant results from the reverse MR were less than the forward MR analysis. One possible reason is that the variance explained by the IVs for IDPs was up to 7.03%, while the variance explained by the IVs for stroke was up to 2.08%. Since we have already used the GWAS data with the largest sample size to date, we cannot add more IVs in our current study. We believe that if more IVs are available in future larger scale GWAS for stroke, more results in the reverse MR analysis might be obtained. We used conservative Bonferroni corrections for multiple testing to keep our results robust. Under such circumstances, although all our MR results can find some similar evidence from previous case-control studies, some previously reported significant causal relationships may not remain in our study. For example, a previous study found that presence of brain infarcts was associated with a smaller hippocampus in elderly participants [51], which were not found in our study. This might because the IDPs we used were subdivided into the left and right hemispheres and that previous study focused on elderly people. In addition, when applied to clinical interventions, the estimated MR effect size must be treated cautiously. The desirable application of predictive results to clinical and health care decisions depends on the effect size of the exposure on the outcome.

## Conclusions

In conclusion, this study used MR to investigate the association between genetically determined IDPs and stroke risk. The findings revealed strong genetic evidence for possible causal links between neuroimaging phenotypes and stroke. This will contribute to better prediction and intervention at the brain-imaging level for the risk of stroke.

## Supplementary Information

**Additional file 1:** **Table S1.** Information for the GWAS summary data used in this study. **Table S2.** Genetic correlation between IDPs and strokes. **Table S3.** Outliers detected by RadialMR in forward MR. **Table S4.** Outliers detected by RadialMR in reverse MR. **Table S5.** IVs associated with confounders in forward MR. **Table S6.** IVs associated with confounders in reverse MR. **Table S7.** The information of IVs for all exposure-outcome pairs in forward MR. **Table S8.** The information of IVs for all exposure-outcome pairs in reverse MR. **Table S9.** Forward MR analysis results. **Table S10.** Reverse MR analysis results. **Table S11.** Pleiotropy test for significant results of the forward MR. **Table S12.** Pleiotropy test for significant results of the reverse MR.

**Additional file 2:** **Figure S1.** Scatter plots for the exposure-outcome pairs with significant inverse-variance weighted (IVW) results in forward MR analysis. **Figure S2.** Scatter plots for the exposure-outcome pairs with significant inverse-variance weighted (IVW) results in reverse MR analysis. **Figure S3.** Leave-one-out analysis plots for traits with significant inverse-variance weighted (IVW) results in forward MR analysis. **Figure S4.** Leave-one-out analysis plots for traits with significant inverse-variance weighted (IVW) results in reverse MR analysis.

## Data Availability

GWAS statistics of brain IDPs were collected from BIG40 web browser (https://open.win.ox.ac.uk/ukbiobank/big40). GWAS statistics of stroke were obtained from MEGASTROKE consortium (https://www.megastroke.org/).

## Abbreviations

AIS: Any ischemic stroke
AS: Including any stroke
CADASIL: Cerebral autosomal dominant arteriopathy with subcortical infarcts and leukoencephalopathy
CES: Cardioembolic stroke
dMRI: Diffusion magnetic resonance imaging
FA: Fractional anisotropy
ICVF: Intracellular volume fraction
IDPs: Imaging-derived phenotypes
ISOVF: Isotropic or free water volume fraction
IVs: Instrumental variables
LAS: Large-artery atherosclerotic stroke
LD: Linkage disequilibrium
LDSC: Linkage disequilibrium score regression
MD: Mean diffusivity
MHC: Human major histocompatibility complex
MO: Diffusion tensor mode
MR: Mendelian randomization
MRI: Magnetic resonance imaging
OD: Orientation dispersion index
OR: Odds ratio
sMRI: Structural magnetic resonance imaging
SVS: Small-vessel stroke
