# Supplementary material for: Assessment of bidirectional relationships between brain imaging-derived phenotypes and stroke: a Mendelian randomization study
Source: BMC Med. 2023 Jul 25;21:271. doi: 10.1186/s12916-023-02982-9 (PMC10369749; doi:10.1186/s12916-023-02982-9)
Supplement: Supplementary file 2 — Additional file 2: Figure S1. Scatter plots for the exposure-outcome pairs with significant inverse-variance weighted (IVW) results in forward MR analysis. Figure S2. Scatter plots for the exposure-outcome pairs with significant inverse-variance weighted (IVW) results in reverse MR analysis. Figure S3. Leave-one-out analysis plots for traits with significant inverse-variance weighted (IVW) results in forward MR analysis. Figure S4. Leave-one-out analysis plots for traits with significant inverse-variance weighted (IVW) results in reverse MR analysis. [file 12916_2023_2982_MOESM2_ESM.pdf]

Supplementary Figure 1

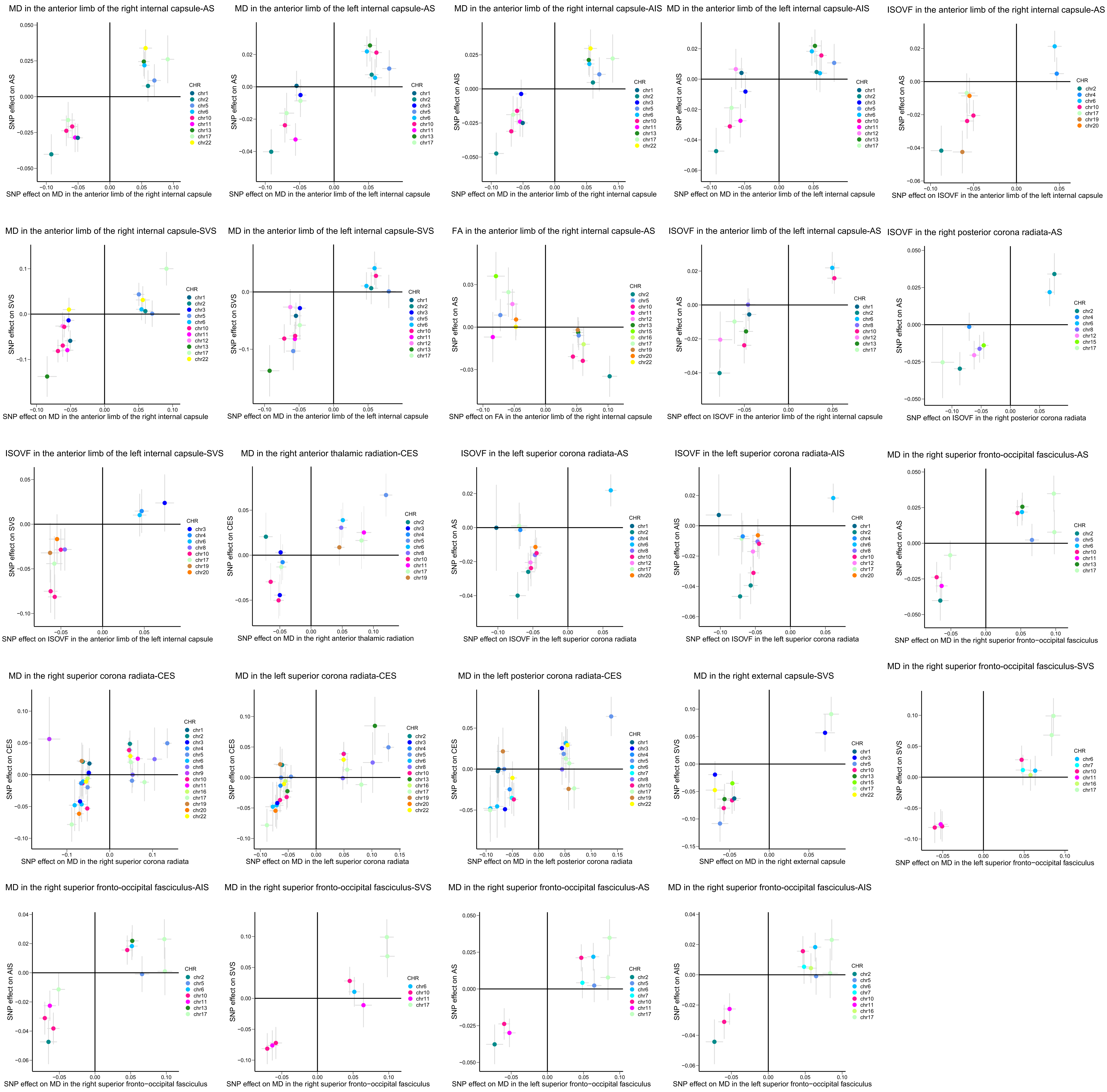

Supplementary Figure 1. Scatter plots for the exposure-outcome pairs with significant inverse-variance weighted (IVW) results in forward MR analysis. Abbreviation: AS, any stroke; AIS, any ischemic stroke; CES, cardioembolic stroke; FA, fractional anisotropy; ISOVF, isotropic volume fraction; MD, mean diffusivity; SVS, small vessel stroke.

Supplementary Figure 2

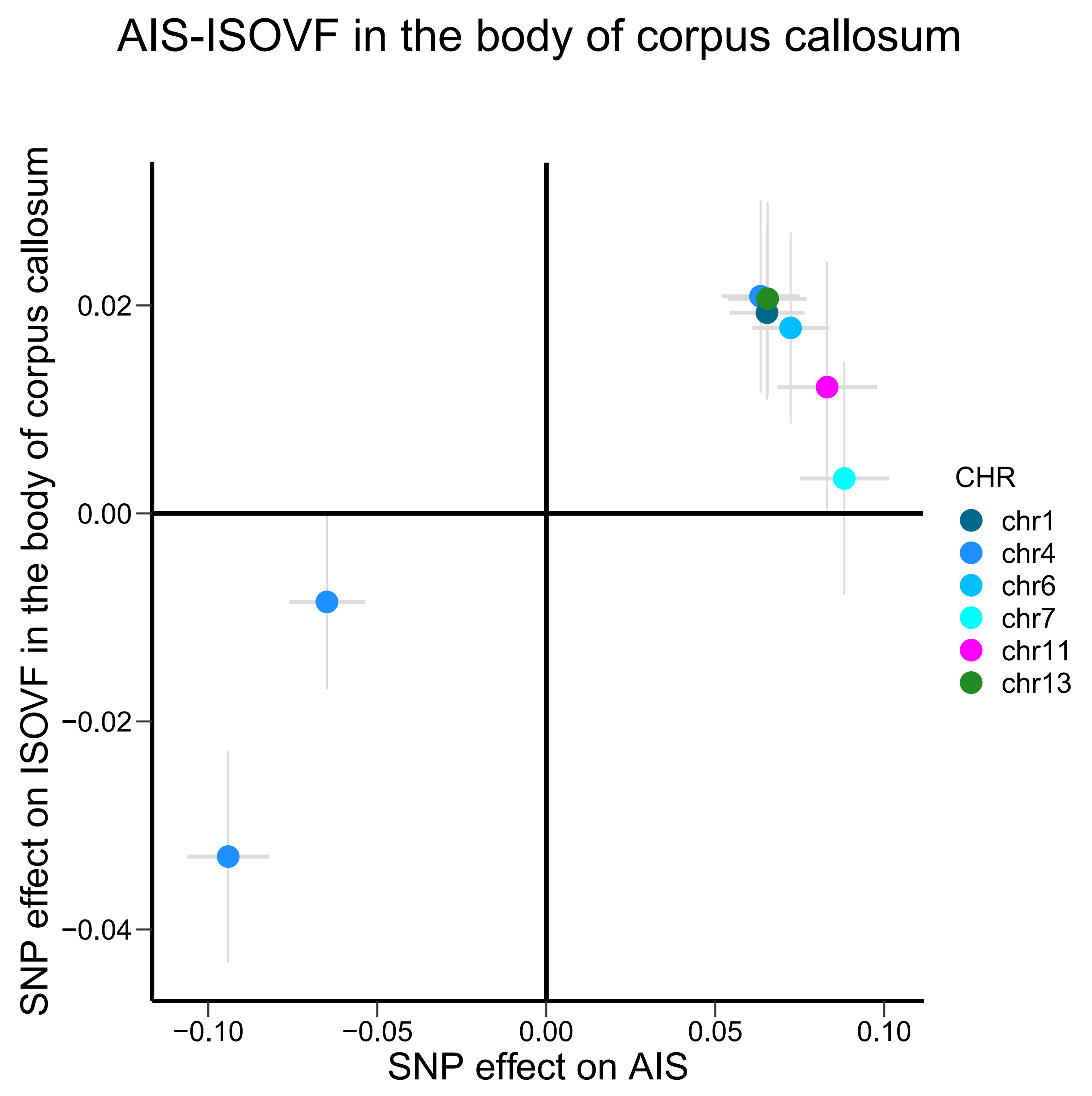

Supplementary Figure 2. Scatter plots for the exposure-outcome pairs with significant inverse-variance weighted (IVW) results in reverse MR analysis.
Abbreviation: AIS, any ischemic stroke; ISOVF, isotropic volume fraction.

Supplementary Figure 3

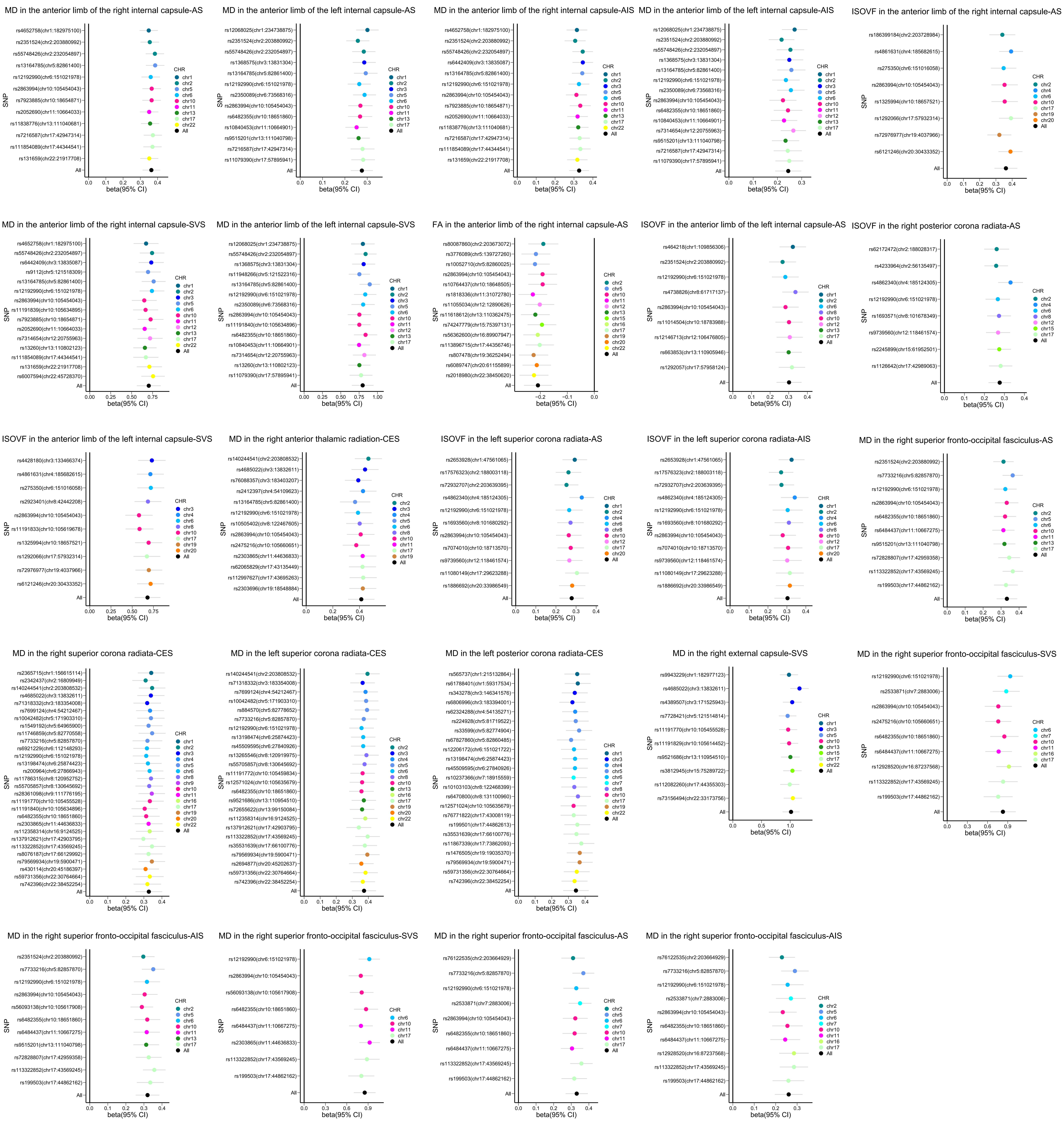

Supplementary Figure 3. Leave-one-out analysis plots for traits with significant inverse-variance weighted (IVW) results in forward MR analysis. Abbreviation: AS, any stroke; AIS, any ischemic stroke; CES, cardioembolic stroke; FA, fractional anisotropy; ISOVF, isotropic volume fraction; MD, mean diffusivity; SVS, small vessel stroke.

Supplementary Figure 4

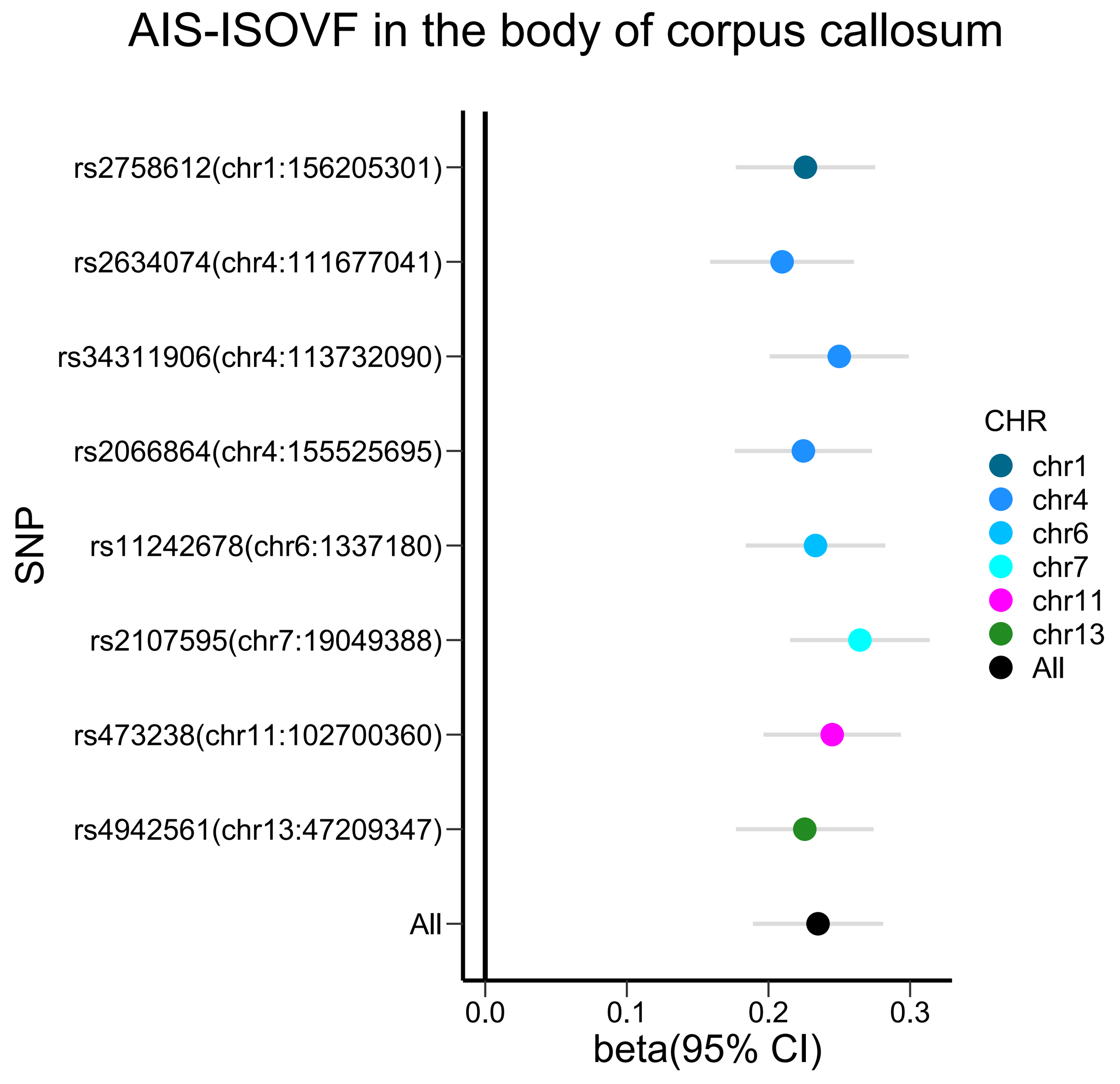

Supplementary Figure 4. Leave-one-out analysis plots for traits with significant inverse-variance weighted (IVW) results in reverse MR analysis.
Abbreviation: AIS, any ischemic stroke; ISOVF, isotropic volume fraction.
